# PyAO: PyTorch-Based Memory-Efficient LLM Training on Ethernet-Interconnected Clusters

**DOI:** 10.3390/s26072269

**Published:** 2026-04-07

**Authors:** Daemin Kim, Hyorim Kim, Juncheol Ahn, Sejin Park

**Affiliations:** Department of Computer Engineering, Keimyung University, Daegu 42601, Republic of Korea; akqjq9250@stu.kmu.ac.kr (D.K.); 5645377@stu.kmu.ac.kr (J.A.)

**Keywords:** distributed training, activation offloading, memory-efficient training, low-bandwidth network

## Abstract

As large language models (LLMs) pursue higher accuracy, their model sizes have surged, substantially increasing GPU memory consumption. Prior work mitigates this issue by distributing the memory burden across multiple GPUs. However, on clusters interconnected via Ethernet, the resulting computational intensity is insufficient to hide the significant network latency. Achieving a favorable compute-to-communication ratio is further constrained by the memory required to cache the massive activations generated during the forward pass. PyAO, proposed in this paper, effectively offloads activations, selects offloading strategies based on their offloading efficiency, and minimizes data-movement bottlenecks, thereby enabling larger micro-batch sizes. In Ethernet-interconnected cluster environments, experiments on popular models—including OPT-1.3B, GPT-0.8B, and Llama-1.2B—demonstrate that PyAO reduces peak GPU memory by up to 1.94× at the same micro-batch size, enables up to 2.5× larger batch sizes, and accelerates training by up to 3.63× relative to the baseline.

## 1. Introduction

The success of large language models (LLMs) across a wide range of applications is largely attributed to their exceptional performance. Because this performance stems from increasingly larger model sizes and higher computational intensity, LLMs have continued to grow in scale [1]. For instance, GPT-2 [2] contained roughly 100 million parameters, whereas recent GPT variants contain hundreds of billions. These massive model sizes inevitably demand substantial memory during training, making it infeasible to run them on a single GPU.

As a result, training such models requires distributing computation and memory across multiple GPUs rather than relying on a single device. In recent years, individuals have equipped their personal machines with high-performance GPUs, enabling fine-tuning or execution of lightweight models as well as compute-intensive workloads such as gaming and photo editing. However, these personal GPUs often remain idle for extended periods, creating a vast pool of underutilized resources globally. This has led to increasing interest in aggregating such idle GPUs into distributed training clusters [3,4,5].

Prior work has explored data parallelism [6], tensor parallelism [7], and pipeline parallelism [8,9] as representative distributed training techniques for scaling both memory and computation. ZeRO [10], built on data parallelism, partitions model states—including optimizer states, gradients, and parameters—across GPUs, thereby reducing the per-device memory requirement from 120 GB to just 1.9 GB.

However, most distributed training systems have been studied primarily in datacenter environments [3] equipped with high-bandwidth interconnects such as InfiniBand [11] and NVLink [12]. Consequently, when deployed on clusters interconnected via Ethernet—providing only 1–10 Gbps of inter-node bandwidth—the network bottleneck can become up to 800× larger compared with InfiniBand configurations that offer 100–800 Gbps (1–8 aggregated links).

The limited bandwidth of Ethernet interconnects leads to significantly lower performance when systems designed for high-bandwidth networks are used in such environments. In Ethernet-connected clusters, network overhead can be mitigated primarily by (1) reducing the frequency of communication and (2) decreasing the volume of data transferred. In this work, we focus on the first strategy. The end-to-end number of communication rounds is proportional to the number of training steps required to reach the target accuracy. Thus, reducing communication rounds requires reducing the step count; in other words, each step must contribute more to accuracy improvement than before. Achieving this necessitates processing a larger amount of data per step, which in turn requires executing more micro-batches.

Moreover, in ZeRO-3, the volume of network communication is fixed at 3M—three times the number of parameter elements (M)—and is therefore independent of the batch size. As a result, increasing the micro-batch size mitigates network bottlenecks by reducing the number of communication rounds without increasing the overall communication volume.

**Figure 1:**
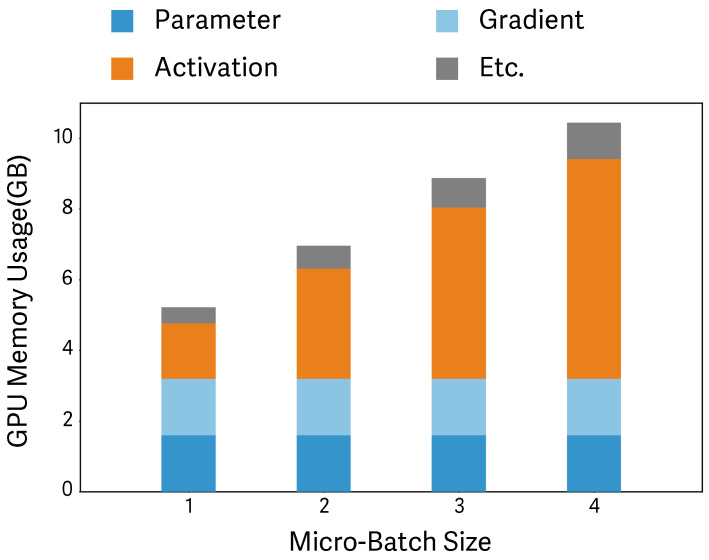
This experiment aims to identify the primary contributors to memory usage across different micro-batch sizes. We evaluate OPT-1.3B on a cluster consisting of five nodes, each equipped with a single RTX 4070 (12 GB). “Etc.” denotes additional temporary buffers.

Increasing the micro-batch size is tightly coupled with memory capacity. Figure 1 illustrates how memory is consumed during training under ZeRO-2. In our experiments, as the micro-batch size increases, total memory consumption rises primarily due to the growth of activations—intermediate tensors cached during the forward pass to compute gradients in the backward pass—rather than model states. Activations account for up to 59% of memory usage, and their proportion increases further with larger micro-batch sizes. Consequently, enlarging the micro-batch size is quickly constrained by available GPU memory.

PyAO, proposed in this paper, is a PyTorch -based framework (PyTorch 2.6.0) designed to maximize the achievable micro-batch size in order to mitigate network bottlenecks in Ethernet-interconnected clusters. To this end, PyAO offloads activations—the dominant contributor to memory consumption—and overlaps computation with CPU–GPU data transfers to reduce transfer overhead. In addition, PyAO reduces peak GPU memory usage during the forward pass through synchronization, minimizes fetch overhead in the backward pass via prefetching, and compresses activations to decrease transfer costs and peak memory usage on both devices. Furthermore, PyAO employs a layer-wise offloading strategy that accounts for heterogeneous offloading efficiency across layers, thereby enabling more effective activation offloading. The key contributions of this paper are as follows:

- We propose **PyAO**, a framework that leverages activation offloading to substantially reduce GPU memory usage, thereby enabling larger micro-batch sizes.
- We optimize activation offloading by overlapping computation with CPU–GPU data transfers, employing prefetching, and compressing activations to reduce transfer overhead. In addition, PyAO adopts a layer-wise offloading strategy based on an offline analysis of per-layer offloading efficiency, excluding layers with low expected benefit to maximize overall efficiency.
- To reduce peak GPU memory usage, PyAO synchronizes computation and data transfers on a per-layer basis during the forward pass and maintains offloaded activations in a quantized form until they are consumed in the backward pass. Activation compression further reduces the size of activations stored in CPU memory, lowering peak CPU memory usage.
- We evaluate PyAO on a cluster interconnected via Ethernet. PyAO reduces peak GPU memory usage by up to 1.94×, enables up to 2.5× more micro-batches per step, and accumulates micro-batches up to 3.63× faster, thereby reducing end-to-end training time. These results demonstrate that PyAO remains highly effective even in bandwidth-constrained environments.

## 2. Background & Motivation

In this section, we describe the core training procedures of LLMs [13,14] and distributed training techniques, and identify the limitations that motivate our work.

### 2.1. Training Pipeline of Large Language Models

Large language models (LLMs) are deep learning models for natural language processing (NLP) built on the Transformer architecture. They are broadly categorized into encoder–decoder, encoder-only, and decoder-only variants. Among these, decoder-only models—despite omitting the encoder—achieve strong performance and have attracted the most attention; this paper likewise focuses on this class. Because LLMs must generate outputs consistent with the given context, they operate autoregressively, predicting the next token conditioned on the input context and previously generated tokens. Training an LLM consists of two major phases: forward propagation and backward propagation.

#### 2.1.1. Forward & Backward Propagation

During the forward pass, given an input sequence $S=(t_{1},t_{2},\ldots ,t_{t},\ldots ,t_{n})$, the model predicts token $t_{t}$ conditioned on the prefix $\left(t_{1},t_{2},\ldots ,t_{t-1}\right)$, while generating predictions for all positions in parallel during training. To predict the next token, computations proceed through all layers of the model, with each layer consuming the output of the previous layer and producing its own output. Throughout this process, activations—the intermediate tensors produced within each layer and required to compute gradients during the backward pass—are cached.

In the final stage of the forward pass, the model computes the training loss by averaging the cross-entropy loss over the probability assigned to the ground-truth token at each position. Let $p_{t}$ denote the predicted probability for the token at position *t*; the objective function is defined as follows:

$$L=-\frac{1}{N}\sum_{t=1}^{N}\log p_{t}$$

During the backward pass, parameter gradients are computed using the loss obtained in the forward pass and the activations cached at each layer. The parameters are then updated with these gradients to minimize the loss.

#### 2.1.2. Activation: The Principal Cause of Memory Exhaustion

The size of cached activations is determined by the batch size, the model’s hidden dimension, and the number of layers, and these activations are primarily stored in GPU memory. As models deepen and batch sizes grow, activation memory increases linearly and becomes the dominant consumer of GPU memory (see Figure 1), ultimately emerging as a key constraint in LLM training. As discussed earlier, efficient training requires executing as many micro-batches as possible per step to minimize network overhead. However, increasing the micro-batch size proportionally increases activation memory, causing training to hit memory limits early. In this paper, we address this activation-induced constraint with an offloading strategy that enables more micro-batches to be executed per step, thereby mitigating network overhead and improving training efficiency.

### 2.2. Distributed Training Techniques

Memory constraints have been a primary limiting factor in the rapid training of large models. To address this, prior work has employed data parallelism (DP), which distributes the training data across GPUs, and tensor parallelism (TP), which splits model parameters within each layer. However, the sequential computation flow of LLMs can introduce idle periods, or bubbles, that lead to underutilization. Pipeline parallelism (PP), which partitions the model into stages and processes micro-batches in a staggered fashion, helps overlap computations and reduce such bubbles, especially when combined with TP. ZeRO, built on data parallelism, has evolved into multiple variants over the past few years [15,16,17].

ZeRO alleviates memory constraints by partitioning model states—optimizer states, gradients, and parameters—across GPUs. Due to its high scalability and ease of integration relative to TP and PP, ZeRO has been widely adopted. It is also more memory-efficient than vanilla DP, which replicates the full model state on every GPU. For these reasons, we adopt ZeRO as the baseline in this work.

#### Increasing Network Overhead on Ethernet-Interconnected Clusters

ZeRO consists of three stages. In ZeRO-1 and ZeRO-2, gradients computed during the backward pass are aggregated and averaged across GPUs via a reduce-scatter, a collective communication operation. In ZeRO-1, each GPU updates the parameters associated with the optimizer states it owns, whereas in ZeRO-2, each GPU updates only the parameters corresponding to its gradient shard. Immediately before the forward pass, an all-gather operation is issued to assemble the updated parameters. Each collective communication call transfers *M* parameter elements, for a total communication volume of 2M.

In ZeRO-3, an all-gather is invoked to assemble the partitioned parameters required for the forward computation, after which the consumed parameter shards are immediately discarded, keeping parameter memory usage consistently low. During the backward pass, the required parameter shards are similarly assembled via an all-gather and discarded after use. In the subsequent optimization step, each GPU performs a reduce-scatter to aggregate the gradient shards for which it is responsible and compute their average. Each communication transfers *M* elements, resulting in a total communication volume of 3M.

As noted earlier, prior studies on distributed training have largely assumed high-speed inter-node interconnects that provide bandwidth one to two orders of magnitude greater than Ethernet (e.g., InfiniBand at 100–800 Gbps versus Ethernet at 1–10 Gbps). Consequently, network overhead becomes significantly more severe on Ethernet-interconnected clusters. Because the per-step communication volume in ZeRO is fixed regardless of the micro-batch size, our approach processes more micro-batches per step to reach the target cumulative number of micro-batches with fewer steps, thereby reducing the total number of communication rounds.

## 3. Problems

This section identifies the key challenges that must be addressed to mitigate communication overhead and accelerate training. Section 4 presents our solutions to each problem.

### 3.1. Memory Exhaustion Caused by Large Activation Data

Because Ethernet-interconnected clusters are severely constrained by communication bandwidth, reaching the target total number of micro-batches quickly requires executing as many micro-batches as possible per step. However, this approach is rapidly limited by the need to cache large activations in finite GPU memory. Thus, activation-induced memory consumption must be alleviated. To address this constraint, we introduce *Activation Offloading*. Since subsequent challenges arise from addressing this primary issue, we present this choice upfront.

### 3.2. Data Transfer Overhead

Offloading activations inevitably introduces additional data-transfer overhead between CPU and GPU memory. This overhead arises from two sources: (1) during the forward pass, computation stalls while activations are offloaded, and (2) during the backward pass, computation stalls while activations are fetched immediately before gradient computation. In both cases, the idle time is approximately the duration of the offload or fetch operation and increases linearly with the activation size, creating a significant bottleneck that can degrade end-to-end training performance.

### 3.3. CPU Memory Pressure from Large Activation Volume

Outside datacenter environments, CPU memory is typically not an order of magnitude larger than GPU memory (e.g., 16 GB, 32 GB, or 64 GB). The commonly used Adam optimizer consumes 12M bytes for optimizer states, exceeding the footprint of parameters (2M) and gradients (2M). Even when partitioned, this nontrivial optimizer-state footprint remains resident on GPUs, which is especially burdensome when GPU memory is limited. ZeRO-Offload provides a mechanism to offload Adam optimizer states to the CPU; accordingly, in our experiments—where GPU memory is constrained (e.g., RTX 4070 with 12 GB)—we also offload optimizer states to CPU memory.

However, freeing GPU memory by offloading optimizer states shifts additional pressure onto CPU memory. Because datasets are already resident in CPU memory, adding optimizer states further reduces the available memory. As a result, the amount of activation data that can be offloaded to the CPU is far from unlimited. Moreover, as the micro-batch size increases, the size of the activations to be offloaded naturally grows, exacerbating both CPU memory pressure and data-transfer overhead.

### 3.4. Layer-Wise Imbalance in Activation Throughput

**Figure 2:**
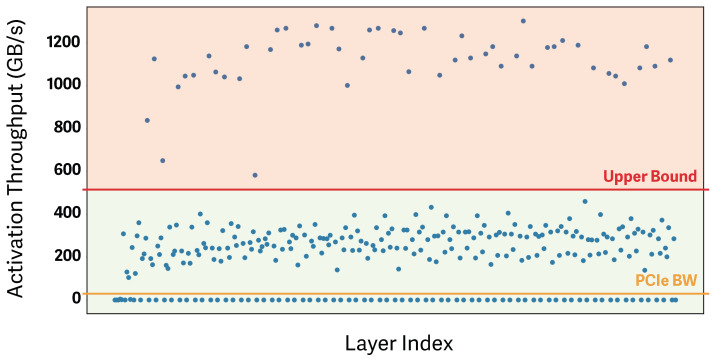
Per-layer offloading efficiency is measured as activation throughput (GB/s). The yellow line denotes the average PCIe bandwidth across nodes—32 GB/s in our setup. The red line represents the cutoff above which the amount of activations produced per second is considered excessive (set to 507 GB/s in our experiments). Accordingly, layers in the green region are considered sufficiently efficient to offload and are included in the strategy, whereas layers in the red region are deemed inefficient and excluded from offloading. Notably, 13% of the layers fall into the red region, which is a substantial fraction given their low offloading efficiency.

Prior work has shown that, in DNNs and LLMs, certain layers emit activations that are disproportionately large relative to their computational cost [14,18]. Figure 2 presents the per-layer activation throughput of GPT-0.8B (GB/s). As illustrated, some layers generate substantially larger volumes of activations per second than others. Because these layers cache high volumes of activations while performing comparatively little computation, the data-transfer cost outweighs the compute cost. Consequently, they introduce bottlenecks due to transfer latency, render naive offloading strategies inefficient, and increase step time. Therefore, indiscriminately offloading all layers is an inefficient approach.

## 4. Design

This section sequentially explains how to address the problems identified in Section 3 and presents the key execution flow of the proposed system, thereby facilitating a comprehensive understanding of the overall design.

### 4.1. Scaling Micro-Batch Size with Activation Offload

Under constrained GPU memory, activation offloading is a commonly employed technique. By moving activations to larger and lower-cost CPU memory, pressure on GPU memory is reduced, enabling more micro-batches to be executed per step. However, while offloading improves memory efficiency, it can stall computation and impose additional pressure on CPU memory. Therefore, offloading must be paired with several optimizations.

### 4.2. Data Transfer Pipeline Optimizations

As noted earlier, activation offloading and fetching can cause computation to stall due to data-transfer latency. To address this, we overlap computation with data transfer so that both can proceed in parallel. As a result, transfer operations no longer block computation, avoiding unnecessary idle time.

Despite this separation, additional issues remain. During the forward pass, naïvely asynchronous offloading can queue transfers such that activations from earlier layers—still in the process of being offloaded—coexist in GPU memory with newly produced activations, thereby increasing peak memory usage. To mitigate this, we synchronize the computation and data-movement streams at the end of each layer. When synchronization completes, no activations remain resident on the GPU, preventing excessive queuing of transfer operations and helping maintain low peak memory.

Even with decoupled streams, the backward pass still incurs fetch overhead because fetching must begin exactly when the corresponding activation is needed for gradient computation. To mitigate this, activations should be fetched onto the GPU ahead of demand. In PyAO, at the start of each layer, we prefetch the activations that will be needed by layers executed later. To ensure data integrity, PyAO synchronizes at the end of each layer, waiting for the prefetch operation to complete so that the data has fully arrived in GPU memory. An exception occurs for the activations produced after the final forward layer: because they are consumed immediately by the subsequent backward computation, offloading and refetching them would be wasteful. Accordingly, we disable the offload/fetch policy for the last layer.

### 4.3. Reducing CPU Memory and Data Transfer Overhead via Activation Compression

Offloading activations to the CPU is comparatively memory-efficient, but CPU capacity is not unlimited. As the micro-batch size increases, the activation footprint grows proportionally, and the memory pressure that appears on the GPU can also emerge on the CPU. To address this, we apply activation compression to relieve CPU-side memory pressure. Compression is performed during the forward pass at the time of caching, before transferring activations to the CPU. Consequently, it reduces CPU memory usage and shortens data-transfer time in both the forward and backward passes by shrinking the transfer payload. During the backward pass, prefetched quantized activations are dequantized immediately before being consumed by computation; this lazy dequantization helps keep peak GPU memory usage low. The compression scheme is detailed in Section 5.

### 4.4. Designing Offloading Strategies Under Heterogeneous Offloading Efficiencies

As shown in Figure 2, offloading efficiency varies across layers, and naively offloading every layer is inefficient (as noted in Section 3.4). Accordingly, PyAO measures per-layer offloading efficiency and excludes low-efficiency layers from the offloading strategy to improve overall effectiveness. To support this, PyAO performs an offline profiling stage that measures, for each layer, the activation throughput produced during the forward pass (GB/s), defined as the cached tensor volume divided by compute time. Layers whose activation throughput exceeds the average PCIe bandwidth across nodes and is identified as an outlier are designated as recompute layers rather than offloaded layers.

In practice, certain layers in Transformer architectures produce disproportionately large activations relative to their computational cost. Our empirical analysis shows that projection layers—which primarily perform dimensionality transformations—and activation-function layers—which generate large activations despite having low arithmetic intensity—are representative examples. These layers exhibit extremely poor offloading efficiency, with activation throughput markedly higher than that of other layers. We therefore treat such layers as statistical outliers and apply the interquartile range (IQR), a widely used outlier-detection metric, to automatically identify them.

Layers whose activation throughput lies above the upper bound are regarded as producing excessive cached activations relative to their computation. These layers are excluded from offloading: all of their activations are discarded during the forward pass and later recomputed during the backward pass to recover the values required for gradient calculation. This removes the data-transfer overhead caused by layers that generate large activations for comparatively little computation, enabling a more efficient offloading strategy at the cost of acceptable recomputation. It also reduces the amount of activation data resident in CPU memory, slightly alleviating CPU-side memory pressure.

### 4.5. PyAO System Execution Sequence

**Figure 3:**
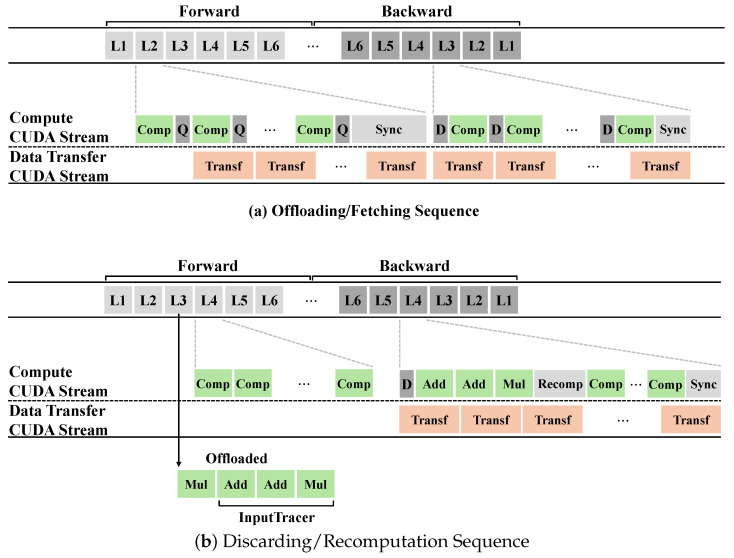
PyAO System Execution Sequence. (**a**) illustrates the offloading timeline, showing when activations are quantized, transferred, and dequantized, as well as when synchronization occurs between the compute and transfer streams. (**b**) illustrates the execution path for layers excluded from the offloading strategy: activations produced during the forward pass are discarded, and during the backward pass, the layer input is reconstructed from the recorded operation trace so that the layer can be recomputed to recover the discarded activations.

This section concludes by visualizing, in Figure 3, the principal execution sequence that demonstrates how offloading operates in conjunction with the optimizations described earlier, and by explaining that sequence in detail.

Figure 3a illustrates when offloading, prefetching, and activation compression are performed. A layer’s computation can be decomposed into a sequence of fine-grained tensor operations. During the forward pass, the output tensors of these operations may be cached for gradient computation during the backward pass; these cached tensors constitute the activations to be offloaded. Before offloading, the activations are compressed and transferred to CPU memory. Because computation and data transfer occur on separate streams, they proceed in parallel without blocking one another. When all tensor operations of a layer complete, PyAO synchronizes the streams to ensure that any ongoing transfers have finished.

During the backward pass, PyAO prefetches the compressed activations required by future layers back into GPU memory. In parallel, gradient computation proceeds. The prefetched activations—already resident on the GPU in compressed form—are decompressed immediately before being consumed. After all layer computations complete, PyAO synchronizes with any remaining prefetch operations to ensure data integrity.

Figure 3b shows the execution sequence for layers excluded from offloading, as described in Section 4.4. These layers are preselected during an offline profiling stage. During the forward pass, because these layers will be recomputed in the backward pass, their inputs must be recoverable. To enable this, PyAO records the operations and parameters of the immediately preceding layer and traces how the current layer’s input was formed. This trace continues recursively until the traced inputs are cached activations; PyAO then records all traced operations and parameter information. All activations produced by this layer during the forward pass are discarded; therefore, no activation compression or transfer is performed for this layer.

During the backward pass, PyAO reconstructs the layer’s input using the recorded trace. To do so, it first dequantizes any prefetched activations required for reconstruction. After reconstructing the input, PyAO recomputes the layer to regenerate the necessary activations and compute gradients. Prefetching for subsequent layers continues concurrently. Because this layer’s activations were not offloaded in the forward pass, the preceding layer in the backward pass performs no prefetch for them, and the current layer incurs no dequantization overhead. Finally, PyAO synchronizes prefetching with computation to ensure data integrity.

## 5. Implementation

This section describes the concrete implementation of the offloading and optimization techniques presented earlier, providing a more detailed view of how PyAO operates in practice.

### 5.1. Hook-Based Activation Offloading

To enable offloading, PyAO identifies activations using PyTorch’s saved_tensors_hooks. The pack hook intercepts activations after they are cached in GPU memory and then offloads them. The unpack hook, invoked during the backward pass, returns the activation required for gradient computation, taking as input the value returned by the pack hook. In our implementation, the pack hook returns a reference to the activation offloaded to CPU memory, allowing the unpack hook to fetch it when needed.

### 5.2. Decoupling Computation and Data Transfer with CUDA Streams

To separate computation from data transfer and enable parallel execution, PyAO assigns distinct CUDA streams to these two classes of work. A CUDA stream queues GPU operations, enforcing in-order execution within a stream while permitting concurrency across streams. With separate streams, data-transfer operations no longer block compute kernels. Because transfers are decoupled from computation, PyAO periodically synchronizes the streams to maintain low peak GPU memory usage and to ensure that data required by subsequent kernels have been fully fetched before they are accessed.

### 5.3. Activation Compression via Lossy Compression

Activation compression can be broadly categorized as lossless or lossy. Lossless compression preserves all information but typically incurs substantial compression and decompression overhead. In contrast, lossy compression introduces some distortion but generally achieves much higher compression ratios with significantly lower overhead. During the forward pass, compression must complete before transfer operations can begin; consequently, heavy compression overhead can stall subsequent transfers. To minimize this impact while still obtaining high compression ratios, PyAO adopts lossy compression. During the backward pass, lossy compression also reduces decompression overhead, allowing prefetched data to become available earlier. Together, these effects shorten end-to-end training time.

For the compression method, we employ symmetric quantization [19,20,21], which offers strong compression with minimal degradation relative to full precision. In our implementation, activations are quantized in the pack hook immediately before offloading, transferred in quantized form between GPU and CPU, and dequantized in the unpack hook immediately before being consumed by computation.

### 5.4. Memory-Efficient Reconstruction and Layer Recomputation

Layers excluded from the offloading strategy are configured such that activations intercepted by the pack hook are discarded rather than stored. However, because these discarded activations must be reconstructed during the backward pass to compute gradients, the corresponding layer must be recomputed, which in turn requires its input. Storing the layer’s input directly would incur additional memory overhead. To avoid this, PyAO traces the operations of the preceding layer and their parameters using PyTorch’s dispatcher for tensor-operation tracing. The trace continues until the inputs to the traced operations are cached activations, enabling reconstruction of the excluded layer’s input without additional memory usage; once traced, the input is discarded. The traced operations and associated parameters are recorded in InputTracer. During the backward pass, this record is used to reconstruct the inputs, allowing the layer to be recomputed and its gradients correctly produced.

### 5.5. An Offline Stage

This section describes how the activation throughput for each layer is computed during the offline stage. Layer-wise throughput is primarily measured using PyTorch Hooks. Specifically, the *pre-forward hook* and *post-forward hook* mark the start and end of each layer’s execution, and the tensors captured by the *pack hook* invoked in between are treated as the activations produced by that layer.

For each interval between consecutive pack hooks, the throughput of that segment is computed by dividing the size of the pack hook’s input tensor by the elapsed execution time. These segment-level throughputs are accumulated over the layer. Once the post-forward hook is triggered, the accumulated throughput is divided by the number of pack-hook invocations to obtain the average throughput for the layer.

This procedure is repeated for approximately 100 steps to stabilize variance. Because the offline stage faithfully replicates the actual training environment and relies on empirical measurement rather than analytical approximation, no additional calibration is required, and the method is agnostic to specific hardware configurations.

## 6. Evaluation

This section compares PyAO with ZeRO-2, a strong baseline that mitigates memory pressure arising from model states. We evaluate peak GPU and CPU memory usage and show that PyAO significantly reduces peak GPU memory at the same micro-batch size. Even under constrained settings, PyAO keeps peak CPU memory within available capacity, demonstrating the feasibility of activation offloading. Finally, we show that PyAO substantially improves end-to-end training time.

### 6.1. Setup

**Settings.** Experiments are conducted on five nodes. Each node is equipped with a single RTX 4070 (12 GB), an Intel i9-13900 CPU, and 64 GB of system memory. All GPUs are connected via PCIe 4.0 ×16 (32 GB/s), and the nodes are interconnected over 1 Gbps Ethernet.

**Baseline.** ZeRO effectively mitigates the static memory footprint associated with model states and is therefore a strong method for distributed training in memory-constrained environments. ZeRO is implemented in the PyTorch-based open-source DeepSpeed framework [22]. Since both PyAO and ZeRO are implemented in PyTorch, ZeRO provides a fair and realistic comparison point. Among the three ZeRO stages, we adopt ZeRO-2 as the baseline. Relative to ZeRO-3, ZeRO-2 incurs approximately 33.3% less inter-node communication (2M vs. 3M elements per step), making it more practical in bandwidth-constrained environments such as Ethernet. At the same time, unlike ZeRO-1, ZeRO-2 shards gradients and achieves additional memory savings. Taken together, these properties make ZeRO-2 the most suitable baseline for our setting.

**Configurations.** We evaluate widely used LLMs with different architectures—OPT [23], GPT-2 [2], and Llama [24]—each with approximately 1 billion parameters (1.3B, 0.8B, and 1.2B, respectively), ensuring that a sufficient number of micro-batches can be executed under our experimental setup. The sequence length is fixed at 512 tokens. Models are trained in half precision, and we use the Adam optimizer [25]. To enable more micro-batches per step, optimizer states are offloaded to CPU memory.

### 6.2. Peak GPU Memory

**Figure 4:**
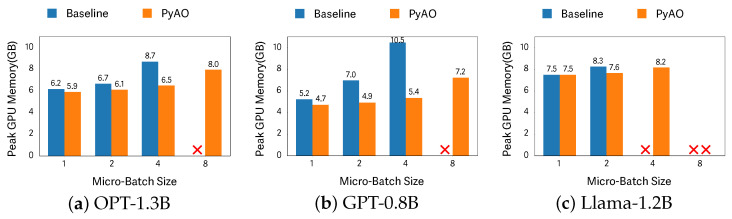
Peak GPU memory per model across micro-batch sizes. “X” denotes an infeasible configuration (e.g., OOM).

Figure 4 illustrates peak GPU memory usage over the training period for each model. At each micro-batch size, both the baseline and PyAO were executed, excluding infeasible configurations. Under the baseline, peak memory increases approximately linearly as the micro-batch size grows, primarily because the activation footprint scales with micro-batch size. Consequently, OPT and GPT-2 cannot be executed at a micro-batch size of 8 due to out-of-memory (OOM) errors, and Llama cannot be executed at micro-batch sizes 4 and 8.

In contrast, with PyAO, peak memory still increases with micro-batch size but at a more moderate rate, because PyAO offloads activations to CPU memory, shifting pressure off the GPU. This reduction in GPU-resident activations lowers peak memory consumption and enables execution at larger micro-batch sizes than the baseline.

For example, with OPT at a micro-batch size of 4, the baseline reaches 8.7 GB, whereas PyAO—at a lower peak memory of 8.0 GB—can execute twice the micro-batch size. For GPT-2 at a micro-batch size of 4, the baseline records 10.5 GB, while PyAO records only 5.4 GB. As a result, the maximum executable micro-batch sizes under PyAO are 10 for both OPT and GPT-2, whereas the baseline limits are 4 and 3, respectively. For Llama, the baseline reaches at most 2, while PyAO reaches 5. Overall, across these experiments, PyAO achieves up to a 1.94× reduction in peak GPU memory and enables micro-batch sizes up to 2.5× larger than those supported by the baseline.

### 6.3. Peak CPU Memory

The optimizer is offloaded to the CPU to mitigate the high GPU memory usage caused by the large Adam optimizer states. Because the optimizer is much lighter than the forward and backward passes, this offloading does not materially degrade throughput. Under mixed precision [26], offloading also allows the full-precision master weights and optimizer states—which have substantially larger footprints—to reside on the CPU rather than the GPU [25]. As a result, a nontrivial portion of the memory burden already rests on the CPU; combined with the footprint of sampled or preloaded datasets, CPU memory can become tight in non-datacenter settings where capacities are modest. Offloading activations to the CPU can therefore create a secondary memory bottleneck, making it essential to keep peak CPU memory usage as low as possible.

**Figure 5:**
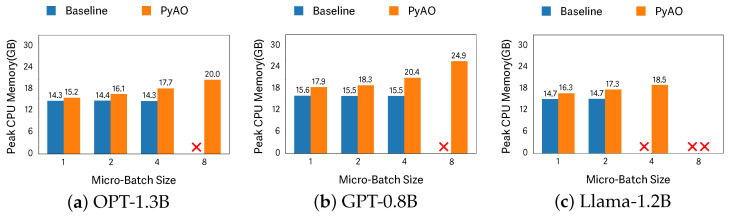
Peak CPU memory per model across micro-batch sizes. “X” denotes an infeasible configuration (e.g., OOM).

Figure 5 shows peak CPU memory usage for the baseline and PyAO across different micro-batch sizes. This experiment evaluates whether activation offloading induces excessive CPU-side memory pressure. Among the configurations executable by both systems, the GPT model at a micro-batch size of 4 exhibits the largest increase (1.32×). This increase is acceptable when weighed against both the substantial GPU-memory burden alleviated by activation offloading and its contribution to training efficiency—namely, enabling more micro-batches per step.

### 6.4. Training Time

From the preceding experiments—conducted under identical settings for both systems—we observe that PyAO enables larger micro-batch sizes than the baseline. This allows each step to process more data and thereby reduces the frequency of network communication, a primary source of slowdown. However, activation offloading can introduce data-transfer overhead that may delay training; PyAO therefore incorporates several optimizations to mitigate these costs. The goal of this section is to show that PyAO not only achieves high memory efficiency but also shortens end-to-end training time.

**Figure 6:**
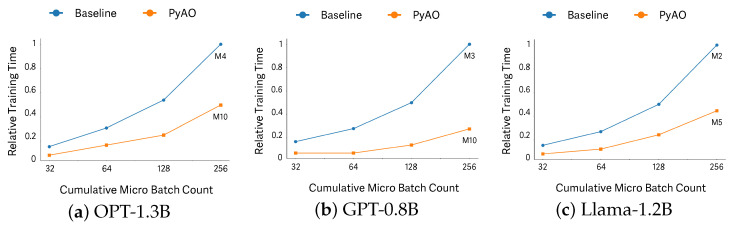
Relative total training time with respect to the accumulated batch count. $M_{x}$ denotes a micro-batch size of *x* (e.g., $M_{10}$ indicates a micro-batch size of 10).

Figure 6 reports the time required for the baseline and PyAO to reach a fixed target of cumulative micro-batches for each model; shorter time indicates higher training throughput. In each run, all GPUs process the same fixed micro-batch size simultaneously, and for each model we select the largest micro-batch size that avoids OOM and remains stable across repeated steps. Under these settings, PyAO reaches the target substantially faster: approximately 2× on OPT, 3.63× on GPT, and 2.24× on Llama (for a target of 256 cumulative micro-batches). These results demonstrate that PyAO’s higher memory efficiency enables larger micro-batches per step, which reduces the number of communication rounds and, in turn, yields up to 3.63× faster time-to-target accumulation.

## 7. Related Works

**Common approaches to reducing activation memory consumption.** Activation checkpointing is a widely used technique for mitigating excessive memory usage arising from activations. However, it discards all intermediate activations and recomputes them when needed during the backward pass. This effectively requires re-executing large portions of the forward pass, increasing wall-clock time and delaying gradient availability, which in turn postpones communication. In contrast, PyAO preserves most activations and applies several optimizations to prevent compute stalls. Only layers with poor offloading efficiency are recomputed, and because their compute cost is small relative to the size of the activations they generate, the associated recomputation overhead is modest.

**ZeRO family of approaches.** ZeRO-Offload [15] applies mixed-precision training and offloads optimizer-related data—including optimizer states, optimizer computations, and FP32 master weights—to the CPU, rather than offloading activation tensors. Thus, the form of activation offloading emphasized in this work is not considered in ZeRO-Offload. Nonetheless, it effectively mitigates the memory overhead introduced by mixed-precision training, and PyAO similarly adopts optimizer offloading. ZeRO-Infinity [16] offloads activation checkpoints that remain after activation checkpointing (i.e., after discarding most intermediate activations) and primarily focuses on optimizing model-state offloading. Consequently, it does not address activation offloading strategies aimed at retaining and reusing most activations, as explored in PyAO. Moreover, ZeRO-Infinity’s evaluations were conducted on DGX-2 nodes interconnected via up to eight InfiniBand links—a high-end setting—leaving its effectiveness in Ethernet-interconnected clusters unverified. ZeRO-R [10] eliminates activation replicas in model-parallel settings and mentions optional activation offloading; however, similar to ZeRO-Infinity, it does not explore offloading optimizations nor provide experimental validation on Ethernet. Finally, ZeRO++ [17] and AMSP [27] primarily target communication bottlenecks and therefore do not reduce the activation memory burden on GPUs.

**Approaches to overcoming communication overhead.** CO_2_ [28] mitigates the network bottleneck—identified as a primary limitation in distributed training—by employing asynchronous all-reduce and local updates. These techniques are compatible with PyAO and could be combined to further accelerate training. StellaTrain [3], similar to PyAO, targets low-bandwidth cluster environments; its asynchronous training scheme and dynamic hyperparameter optimization are likewise complementary and may enable more efficient training on bandwidth-constrained clusters when integrated with PyAO.

**Memory-Managements within Activation Offloading** [29] formulates the cost of offloading, checkpointing, deleting, and backward operations in terms of time and memory consumption, and uses dynamic programming to determine optimal decisions—such as how to make a decision in segment-wise during the forward pass and when to prefetch during the backward pass. Because this approach searches for an optimal schedule at runtime, it incurs online scheduling overhead during training. In contrast, PyAO determines its offloading strategy entirely in an offline stage, enabling efficient offloading during training without introducing additional runtime overhead.

ProTrain [30] proposes a block-wise activation management scheme that adapts activation offloading decisions at runtime based on buffer usage, I/O bandwidth, and memory availability. However, ProTrain also relies on online decision-making and therefore does not benefit from the advantages of an offline-planned strategy.

SSDTrain [31] schedules activation offloading and prefetching across SSD, CPU, and GPU memory on a per-layer basis. However, because it just offloads all activations, it misses opportunities to leverage recomputation for layers where recomputation would be more efficient.

In contrast, PyAO analyzes recomputation efficiency during its offline stage and selectively avoids offloading layers where recomputation is significantly cheaper, resulting in a more efficient and hardware-aware offloading strategy.

## 8. Discussion

This section discusses several important topics that were not covered earlier but are essential for evaluating PyAO’s practicality. We analyze PyAO’s behavior under CPU-memory constraints—a critical but often overlooked factor—and outline promising directions for future work.

### 8.1. Worst-Case CPU Memory Usage

Most prior studies focus primarily on reducing GPU memory usage; however, in general-purpose PC environments, CPU memory is also limited (e.g., 16, 32, or 64 GB). Therefore, CPU memory cannot be treated as an unlimited resource, and any offloading-based approach must carefully consider whether it remains feasible under constrained CPU capacity. In this section, we examine whether PyAO can still operate reliably under the worst-case CPU-memory scenario.

The major contributors to CPU memory usage in PyAO are the optimizer state, FP32 model parameters, loaded datasets, and activations/buffers.

First, because PyAO employs optimizer offloading, FP32 optimizer states reside in CPU memory. Since ZeRO-2 is used, these states are additionally partitioned across nodes. The FP32 model parameters needed for optimizer updates also remain on the CPU. Although datasets are very large in total size, PyTorch uses an on-the-fly loading mechanism that reduces CPU-memory pressure. Only a small portion of the data is kept in memory at once, and upcoming samples are swapped in as needed. Thus, the dataset itself contributes little to overall CPU memory usage.

Activations grow linearly with the amount of training data and are, as discussed earlier, the dominant source of memory usage during training. This is also reflected in PyAO, where activations constitute the major CPU-memory component. Detailed memory usage is shown in Table 1.

In PyAO, the sizes of the optimizer state and model parameters may grow depending on model scale, but the size of the model that can practically be executed is inherently limited by system capacity. The model used in our experiments contains 1B parameters, which is an appropriate size that allows PyAO to run with a micro-batch size that is neither too small nor excessively large for our environment.

Given a fixed model size, the remaining factor that grows linearly with input size is the activation memory. Under the configuration that supports the largest feasible micro-batch size without GPU OOM, PyAO consumed 28.66 GB of CPU memory (conducted on the OPT-1.3B, micro-batch size = 8, sequence-length = 512, 5 nodes). This is well below the system’s 64 GB limit and is still manageable even on a 32 GB machine. Therefore, PyAO remains fully executable even under worst-case CPU-memory constraints and can operate safely in typical memory-limited environments.

### 8.2. CPU Bandwidth Contention

CPU bandwidth contention is also an essential issue that any offloading-based system must consider, and thus we examine potential contention scenarios in PyAO. The main data transfers between the CPU and GPU involve gradients and activations. Activations are written from the GPU to the CPU during the forward pass for offloading, and read back during the backward pass for prefetching. Gradients generated during backward propagation are immediately offloaded to the CPU, and once enough gradient shards accumulate in the buffer, an all-reduce communication is triggered.

For clarity, we describe memory movement in two stages. During the forward pass, only activations are transferred, and this corresponds to GPU-to-CPU writes. Because only one type of transfer occurs, there is no contention, and the synchronization overhead between compute and transfer streams remains small for each layer (see Table 1).

During the backward pass, activation prefetching results in CPU reads, gradient offloading results in CPU writes, and once enough gradient shards accumulate, CPU reads also occur for all-reduce. Although PCIe supports bidirectional transfer without contention, these three CPU memory operations contend for DRAM bandwidth and can significantly delay execution.

A typical solution to such contention is to use NUMA-aware scheduling, where conflicting tasks are distributed across different NUMA nodes so that each task accesses local memory and contention is reduced. However, PyAO assumes consumer-grade GPUs and general-purpose PCs, where NUMA-based solutions are less applicable.

Another possible solution is priority-based dynamic scheduling. Tasks have different priorities depending on the training phase. Among activation reads, gradient writes, and gradient reads in the backward pass, gradient reads are the most critical because they directly affect inter-node communication, and delaying them introduces idle time on the network. The relative priorities of the other two operations also depend on the situation. For example, when activation prefetching is not immediately needed in backward operation, prioritizing gradient writes allows partial all-reduce to proceed more quickly. Conversely, if computation is about to start and the required activation has not yet been fetched, activation reads should be prioritized.

Such prioritization must dynamically account for bandwidth and when each piece of data is required by computation or communication. Designing a dynamic scheduler that mitigates contention in this manner is an important direction, and we leave it for future work.

### 8.3. Is the Offloading Strategy Efficient?

PyAO derives its offloading policy in an offline stage by excluding a subset of layers. These excluded layers are those whose activation sizes are disproportionately large relative to their computation cost. Such layers are designated for recomputation, and excluding them is expected to enable a more efficient offloading schedule.

In this section, we quantitatively evaluate whether the resulting offloading strategy is indeed effective and whether it yields a measurable speedup in training.

Before presenting the experiments, we note that in our experimental environment, the inter-node communication bandwidth is limited to 1 Gbps, causing communication time to be roughly an order of magnitude larger than non-communication time. Due to this imbalance, improvements in PyAO’s activation-offloading pipeline are not prominently reflected in the end-to-end results; therefore, our analysis focuses on the non-communication components. If the network bandwidth were even 10 Gbps, communication cost would decrease dramatically, and the benefits of PyAO would become clearly visible even when communication time is included—yielding substantially larger practical gains.

We measure the average step time in two settings: (1) offloading applied to all layers, and (2) offloading applied according to the offline-derived strategy. We further break down each component of the step time to provide a fine-grained analysis.

In the configuration where all layers are offloaded, we first measure synchronization overhead in the forward pass, which arises from pipeline bubbles between the compute stream and the data-transfer stream, i.e., offloading latency that cannot be fully overlapped. Compression overhead is also measured.

During the backward pass, since prefetching is used and synchronization is enforced at the end of every layer, we measure these synchronization stalls as well. The cost of prefetching reflects the time spent fetching activations back to the GPU. Decompression overhead is also reported.

Next, we evaluate the setting where offloading is applied only to layers selected during the offline profiling stage. In the forward pass, we measure synchronization overhead, compression overhead, and the cost of tracing inputs for recomputation. In the backward pass, we measure synchronization at each layer boundary, the time to restore inputs, as well as the cost of recomputing activations through partial forward recalculation. Decompression overhead is also included.

Table 2 show that when all layers are offloaded, synchronization overhead in both forward and backward passes is substantially larger than in the recomputation-based configuration. This is expected because large activations—those that should have been recomputed—are instead offloaded and fetched. The effect is even more pronounced in the backward pass due to additional CPU-memory bandwidth contention.

Similarly, compression and decompression overheads increase when large activations are transferred instead of recomputed. In contrast, the total recomputation cost (forward + backward) incurred under the selective-recompute strategy is smaller than the synchronization stalls observed when offloading all layers.

As a result, applying offloading to all layers—without the offline selection stage—increases step time by approximately 7%, confirming that selective recomputation yields a meaningful performance improvement. This performance gap is expected to widen further under lower PCIe bandwidths, longer sequence lengths, or larger model scales.

### 8.4. Does Two Stream Work Even with Consumer-Grade GPU?

Consumer-grade GPUs often provide only a single copy engine. In such cases, data transfers between the device and host cannot be executed in parallel. However, as discussed in Section 8.2, the forward pass issues only one type of transfer, allowing two CUDA streams to fully exploit compute–communication overlap. In contrast, during the backward pass, activation fetching and gradient offloading cannot be processed simultaneously, which delays data movement. This explains why the synchronization time in Table 2 is noticeably higher during the backward pass than during the forward pass. Even so, overlapping compute and communication is still more efficient than executing them sequentially with computation. As shown in Table 3, using two streams reduces the step time by approximately 19%. Ultimately, the compute–communication overlap enabled by two streams remains effective and meaningful even on consumer-grade GPUs equipped with a single copy engine.

### 8.5. Memory Managements for PyAO

In systems that rely on offloading, such as PyAO, paging overhead can have an even more significant impact on training time. To avoid delays caused by paging, pinned memory is commonly used, and PyAO makes extensive use of it as well. However, allocating pinned memory incurs some overhead, and frequent deallocation and reallocation can lead to fragmentation. To avoid this, PyAO reuses pinned memory throughout the entire training process rather than repeatedly allocating and freeing it. Although retaining memory without releasing it may raise concerns about excessive memory usage, the pinned memory reserved for offloading has a fixed size and is continuously utilized during training, so this does not pose a practical problem.

### 8.6. Compression Error

PyAO applies activation compression within the offloading pipeline and operates alongside mixed precision. Although mixed precision reduces casting errors through scaling, it still introduces information loss. In addition, symmetric quantization also incurs errors during the scaling process. These sources of error overlap, but this paper does not provide a detailed analysis of how such cumulative error may affect final accuracy.

The reason is that the primary objective of this work is to maximize throughput under constrained network conditions. Activation compression in PyAO is introduced as an optional mechanism to reduce data-transfer volume when PCIe bandwidth is limited or when CPU-side contention becomes a bottleneck, thereby improving overall throughput. In other words, compression is an optional enhancement motivated by system constraints, and the paper focuses on demonstrating how an efficient pipeline can be designed and implemented when activation compression is enabled.

For this reason, the paper emphasizes throughput results like [29,30,31] and pipeline behavior rather than accuracy or error analyses, even when activation compression and mixed precision are used together. Nevertheless, the authors acknowledge that studying the accuracy implications is important. Mixed precision combined with automatic loss scaling is known to mitigate numerical error effectively [26,32], but there is still limited research on how mixed precision interacts with additional quantization error when both are used simultaneously.

Since activation compression broadens the applicability of activation offloading in diverse environments, this area of investigation is meaningful and important. The authors therefore leave a deeper analysis of numerical error and model accuracy under combined mixed precision and activation compression as future work.

### 8.7. Integration of PyAO with Emerging Peer-to-Peer Distributed Training Frameworks

Recent advances have introduced peer-to-peer-based distributed training frameworks, many of which are compatible with PyAO. These approaches can be combined to build even more powerful and scalable systems. In this section, we discuss how such integrations can be achieved at a high level.

We first consider ATOM [4], where each process stores a full model replica in CPU memory and performs profiling to measure computation time, memory usage, and loading overhead, thereby determining when to prefetch or swap out model blocks. As discussed in PyAO’s offline stage, certain layers exhibit disproportionately large activation sizes relative to their computation cost, and in some cases, the activation footprint may even exceed the size of the layer’s parameters. Consequently, ATOM’s scheduling must account for the additional cost of activation offloading and recomputation at the layer level.

As the number of scheduling factors increases, the overhead of generating an optimal schedule naturally grows. However, if this overhead can be reduced through profiling-informed heuristics or lightweight cost models, a combined ATOM–PyAO system could yield a more memory-efficient and faster architecture.

Next, FusionAI [5] constructs a DAG representing forward, backward, and optimizer operators, using nodes to represent operators and edges to encode dependencies. Subgraphs of this DAG are dynamically assigned to heterogeneous nodes, while a broker and backup pool enhance robustness. All communications are performed through a DHT-based peer-to-peer mechanism, providing decentralization and improved privacy.

FusionAI accounts for heterogeneous compute resources, which PyAO does not explicitly model, and its ability to tolerate node churn increases practical effectiveness. However, even with heterogeneous DAG partitioning, large-scale distributed training inevitably drives GPU memory pressure as models and datasets grow. In such scenarios, PyAO can alleviate per-node memory constraints through selective activation offloading and recomputation, enabling FusionAI to operate efficiently even when individual nodes have limited GPU memory.

### 8.8. LLMs in Network-Centric and Distributed Environments

Recent work has explored the use of LLMs for network-centric applications, particularly in distributed and wide-area settings [33,34]. While these studies demonstrate the applicability of LLMs to networking tasks, they largely abstract away the system-level challenges of training large models under limited bandwidth and memory constraints. PyAO addresses this gap by enabling efficient LLM training over low-bandwidth network environments through selective activation offloading and recomputation, thereby providing a practical foundation for future network-oriented LLM systems.

## 9. Future Works

As discussed in Section 8.6, our future goals include reducing the overhead of activation compression, minimizing information loss to preserve model accuracy, and introducing a priority-based dynamic scheduling mechanism to mitigate CPU bandwidth contention.

Beyond the 1 Gbps Ethernet environment used in this work, we aim to extend PyAO to WAN-scale distributed training setups, including heterogeneous GPU clusters operating under even more constrained network conditions.

As future work, we leave the design of a system that integrates blockchain into WAN-based clusters to provide trustworthy inter-node communication and to prevent malicious gradient tampering that could degrade training.

More broadly, cross-domain advances in signal optimization and noise-aware data processing, including recent work in sensing systems [35], may provide useful inspiration for future compression-aware training pipelines.

## 10. Conclusions

We propose PyAO, a PyTorch-based framework for memory-efficient distributed training. By applying activation offloading effectively, PyAO reduces peak GPU memory usage by up to 1.94×, enabling up to 2.5× more micro-batches per step; the resulting increase in CPU memory usage is at most 1.32×, a reasonable trade-off given the benefits of offloading. In addition, PyAO incorporates optimizations that mitigate the delays introduced by activation offloading, achieving up to 3.63× faster training time than the baseline. All experiments were conducted on a 1 Gbps Ethernet–interconnected cluster without high-speed interconnects, demonstrating that PyAO remains effective in low-bandwidth environments.

## Figures and Tables

**Table 1:** CPU memory usage breakdown for different model configurations in PyAO. Memory usages are reported in GB. This experiments were conducted with a micro-batch size of 1 and a sequence length of 512.

Model	Parameters	Optimizer States	Datasets	Activation + Buffers	Total
OPT-1.3B	5.28	2.12	0.20	6.70	14.30
GPT-0.8B	4.92	1.97	0.20	10.81	17.90
Llama-1.2B	6.50	2.60	0.20	7.00	16.30

**Table 2:** Breakdown of the average step execution time for three configurations: a single-stream offloading baseline, PyAO without recomputation, and the proposed PyAO. Times are reported in seconds. Experiments were conducted using the OPT-1.3B model with a micro-batch size of 8 and a sequence length of 512. The comparison excludes network time, which is approximately 36 s for these methods.

Method	Forward				Backward					Optimizer	Total Step
	Comp.	Sync.	Compr.	Inp. Trace	Comp.	Fetch	Inp. Recomp.	Act. Recomp.	Decompr.	Comp.	Time
One Stream Off.	2.5573	–	–	–	1.8608	–	–	–	–	0.1673	4.5856
PyAO w/o Recomp.	1.0518	0.0213	0.3949	–	1.4531	0.1451	–	–	0.0811	0.1663	3.3135
PyAO	1.0137	0.0005	0.3818	0.0002	1.4680	0.0197	0.0003	0.0044	0.0442	0.1663	3.0998

**Table 3:** Comparison of training step time with one vs. two CUDA streams. A total of 100 iterations were executed, and both prefetching and quantization were disabled to clearly evaluate how introducing a second stream improves compute–communication overlap and, consequently, offloading efficiency. This experiment was conducted on the OPT-1.3B model with a micro-batch size of 8 and a sequence length of 512 within single node.

Model	Number of Streams	Batch Size	Avg. Step Time
OPT-1.3B	One	8	4.59 s
	Two	8	**3.72 s**

## Data Availability

The code implemented and executed in this paper can be found here https://github.com/Yeosu-expo/PyAO (accessed on 10 December 2025).
